# Dual mode of action of Bt proteins: protoxin efficacy against resistant insects

**DOI:** 10.1038/srep15107

**Published:** 2015-10-12

**Authors:** Bruce E. Tabashnik, Min Zhang, Jeffrey A. Fabrick, Yidong Wu, Meijing Gao, Fangneng Huang, Jizhen Wei, Jie Zhang, Alexander Yelich, Gopalan C. Unnithan, Alejandra Bravo, Mario Soberón, Yves Carrière, Xianchun Li

**Affiliations:** 1 Department of Entomology, University of Arizona, Tucson, AZ, 85721, USA; 2 U.S. Department of Agriculture, Agricultural Research Service, U.S. Arid Land Agricultural Research Center, Maricopa, AZ 85138, USA; 3 Department of Entomology, College of Plant Protection, Nanjing Agricultural University, Nanjing 210095, China; 4 Department of Entomology, Louisiana State University Agricultural Center, Baton Rouge, LA 70803, USA; 5 State Key Laboratory for Biology of Plant Diseases and Insect Pests, Institute of Plant Protection, Chinese Academy of Agricultural Sciences, Beijing 100026, China; 6 Instituto de Biotecnología, Universidad Nacional Autónoma de México, Cuernavaca 62250, Morelos, Mexico

## Abstract

Transgenic crops that produce *Bacillus thuringiensis* (Bt) proteins for pest control are grown extensively, but insect adaptation can reduce their effectiveness. Established mode of action models assert that Bt proteins Cry1Ab and Cry1Ac are produced as inactive protoxins that require conversion to a smaller activated form to exert toxicity. However, contrary to this widely accepted paradigm, we report evidence from seven resistant strains of three major crop pests showing that Cry1Ab and Cry1Ac protoxins were generally more potent than the corresponding activated toxins. Moreover, resistance was higher to activated toxins than protoxins in eight of nine cases evaluated in this study. These data and previously reported results support a new model in which protoxins and activated toxins kill insects via different pathways. Recognizing that protoxins can be more potent than activated toxins against resistant insects may help to enhance and sustain the efficacy of transgenic Bt crops.

Insecticidal proteins from the common soil bacterium *Bacillus thuringiensis* (Bt) are used extensively in sprays and transgenic plants to control insects that attack crops and vector diseases^1^,^2^. These Bt proteins are especially valuable because they kill some of the world’s most harmful pests, yet are not toxic to people and most other organisms^2^,^3^,^4^,^5^. Global planting of crops genetically engineered to produce Bt proteins increased to 78 million hectares in 2014, with a cumulative total of 648 million hectares since 1996^1^. Although Bt sprays and Bt crops have provided substantial economic and environmental benefits^1^,^2^,^6^,^7^,^8^,^9^, rapid evolution of pest resistance to Bt proteins is eroding these advantages^10^,^11^,^12^,^13^,^14^.

Understanding the mode of action of Bt proteins is critical for enhancing and sustaining their efficacy against pests. In particular, many studies have examined the mode of action of the crystalline Bt proteins Cry1Ab and Cry1Ac, which kill lepidopteran pests and are produced by widely adopted transgenic Bt corn, cotton, and soybean^1^,^2^,^15^,^16^,^17^,^18^. All models of Bt mode of action agree that the full-length forms of Cry1Ab and Cry1Ac proteins called protoxins are converted by insect midgut proteases to activated toxins that bind to insect midgut receptors (Fig. 1)^15^,^16^,^17^,^18^. This activation entails removal of approximately 40 amino acids from the amino terminus and 500 amino acids from the carboxyl terminus, converting the protoxins of approximately 130 kDa to activated toxins of approximately 65 kDa^15^,^16^,^17^,^18^. Although competing models differ in post-binding events that eventually kill insects, the currently accepted paradigm asserts that protoxins do not bind to midgut receptors and must be converted to activated toxins of approximately 65 kDa to bind to larval midgut receptors and exert toxic effects^15^,^16^,^17^,^18^.

Contrary to this paradigm, however, *in vitro* experiments with *Pectinophora gossypiella* showed that both the protoxin and activated toxin forms of Cry1Ac bind to fragments of cadherin, a key midgut receptor protein, and to brush border membrane vesicles prepared from insect midguts^19^. These unexpected results raised the intriguing possibility that binding of protoxins to midgut receptors can kill insects via a toxic pathway different from the primary pathway initiated by binding of activated toxins to midgut receptors.

Crystallography recently revealed that, like activated toxins, the carboxyl half of Cry1Ac protoxin that is removed during activation is organized into distinct structural domains^20^ (Fig. 1). Domains V and VII of this portion of Cry1Ac protoxin resemble carbohydrate-binding modules and are structurally similar to domains II and III of the activated toxin^20^ that mediate binding to midgut receptors^16^,^17^. In addition, recent *in vitro* experiments showed that both the protoxin and activated toxin forms of Cry1Ab bind to the same cadherin fragment from *Manduca sexta*, with only slightly lower binding affinity for protoxin relative to activated toxin^21^. Moreover, both forms promoted post-binding events in the toxic pathway, including oligomerization and pore formation, with one oligomer formed by protoxin and a different oligomer formed by activated toxin^21^.

Based primarily on the *in vitro* findings summarized above, Gomez *et al.*^21^ proposed a new model for Bt mode of action, which we refer to here as the “dual model,” where both the protoxin and activated toxin forms can kill insects, with each form exerting its toxic effect via a different pathway (Fig. 1). This contrasts with what we refer to hereafter as the “classical model” described above in which protoxins are inactive. The data cited above that spurred the dual model are valuable because the *in vitro* approach eliminated activation of protoxins by proteases in the insect midgut. However, experiments *in vivo* are essential to determine which model more accurately describes what happens inside live insects.

Here we test the classical and dual models using nine sets of bioassays that compare responses to protoxins versus activated toxins against seven resistant strains and three susceptible strains of three major pests (*Helicoverpa armigera, Helicoverpa zea*, and *Diatraea saccharalis*) from two families of Lepidoptera (Noctuidae and Crambidae) (n = 11,520 insects tested in 34 bioassays, Tables S1–S5). The results show that protoxins were generally more potent than activated toxins against resistant strains, which contradicts the classical model and supports the dual model.

## Results

### Resistance lower to protoxins than activated toxins

According to the classical model, protoxins must be converted to activated toxins to kill insects, and disruption of this step causes higher resistance to protoxins than to activated toxins^22^,^23^,^24^,^25^,^26^. The classical model also predicts that reduced binding of activated toxins to midgut receptors, the most common and most potent mechanism of resistance, causes similar resistance to protoxins and activated toxins^27^,^28^. Thus, the classical model predicts that resistance will not be lower to protoxins than activated toxins.

Contrary to this prediction, resistance to Cry1Ab or Cry1Ac was lower to protoxins than the corresponding activated toxins in eight of the nine new data sets reported in this study (Fig. 2, Tables S2–S4). We calculated the resistance ratio as the concentration of activated toxin (or protoxin) killing 50% of larvae (LC_50_) for a resistant strain divided by the LC_50_ of activated toxin (or protoxin) for a conspecific susceptible strain. We measured the reduction in resistance ratio for the protoxin relative to its activated toxin as the resistance ratio for the activated toxin divided by the resistance ratio for the corresponding protoxin. For the nine cases, the median resistance ratio was 140 for activated toxins versus 21 for protoxins, with a median 10-fold reduction in resistance ratio for protoxins relative to activated toxins (paired t-test of log-transformed data, t = 4.9, df = 8, P = 0.001).

In the sole exception to the pattern of higher resistance ratios for activated toxin than protoxin in the nine cases from this study, the resistance ratio was 7.9 for trypsin-activated Cry1Ac and 17 for Cry1Ac protoxin against the GA strain of *H. zea*. GA was derived from a population that was exposed to Bt proteins in the field^29^, but had been reared subsequently in the laboratory for more than 40 generations without exposure to Bt proteins and was the least resistant of the seven resistant strains tested here (Fig. 2, Table S4). For the GA-R strain, which was derived from GA and selected with Cry1Ac protoxin in the laboratory^29^, the resistance ratio was 82 for trypsin-activated Cry1Ac versus 6.4 for Cry1Ac protoxin (Table S4). For both GA and GA-R, resistance to Cry1Ac protoxin was lower than resistance to Cry1Ac activated with midgut juice from a susceptible strain of *H. zea* (Fig. 2, Table S4). Across all nine cases, the reduction in resistance ratio for protoxin relative to activated toxin increased significantly as the resistance ratio for activated toxin increased (regression of log-transformed data, r^2^ = 0.71, df = 7, P = 0.004, Figure S1).

### Potency of protoxins relative to activated toxins

Based on the assumption that protoxins must be converted to activated toxins to kill insects, the classical model predicts that activated toxins will be more potent than their protoxin counterparts. More specifically, it has been hypothesized that Cry1A protoxins will be about half as potent as their activated toxins, because the active portion of these protoxins weighs about half (65 kDa) of the total of the protoxin (130 kDa)^30^. To test this hypothesis, we evaluated potency, which is inversely related to the LC_50_ value^31^. We calculated the potency of protoxin relative to activated toxin as the LC_50_ of the activated toxin divided by the LC_50_ of the corresponding protoxin^31^.

The results from analyzing potency for the new data reported here are consistent with the classical model for susceptible strains, but not for resistant strains (Fig. 3, Table S5). Against susceptible strains, the mean potency of protoxins relative to activated toxins was 0.70 (range = 0.17 to 1.6), which does not differ significantly from the predicted value of 0.50 (one sample t-test of log-transformed data, df = 7, t = 1.5, P = 0.18). However, contrary to the classical model, protoxins were more potent than activated toxins in eight of nine pairwise comparisons for resistant strains (Fig. 3, Table S5). The lone exception again involved the GA strain tested with trypsin-activated Cry1Ac (potency of protoxin relative to activated toxin = 0.72). Nonetheless, consistent with the overall trend, Cry1Ac protoxin was 4.3 times more potent than Cry1Ac activated with midgut juice against this strain (Fig. 3, Table S5). Against all resistant strains, the mean potency of protoxins relative to activated toxins was 5.7 (range = 0.72 to 39), which is more than 10 times greater than the predicted value of 0.50 (one-sample t-test of log-transformed data, df = 8, t = 6.8, P = 0.0001). Moreover, the potency of protoxins relative to activated toxins was significantly greater for resistant strains than susceptible strains (paired t-test of log-transformed data, df = 8, t = 4.8, P = 0.001).

## Discussion

The responses to Cry1A protoxins and activated toxins in bioassays with 10 resistant and susceptible strains of three species reported here generally support the dual model, but not the classical model. Previous results from five additional resistant and susceptible strains of two species also support the dual model, but not the classical model. Similar to our findings, the resistance ratio was higher for activated toxin than protoxin for the Europe-R and RSTT-R strains of *Ostrinia nubilalis*^32^. The resistance ratio for Cry1Ab was 108 for activated toxin versus 5.7 for protoxin (a 19-fold difference) for Europe-R; and 484 for activated toxin versus 15 for protoxin (a 32-fold difference) for RSTT-R^32^. Also similar to the results reported here, Cry1Ab protoxin was less potent than activated toxin against a susceptible strain (relative potency = 0.27), but the protoxin was more potent than activated toxin against both resistant strains (relative potency = 13 for Europe-R and 15 for RSTT-R)^32^. Likewise, results with the resistant AR strain of *H. zea* show higher resistance to activated toxin than protoxin, as well as greater potency of protoxin than activated toxin against the resistant strain^30^.

Considering the new data reported here together with previous results, evidence from 10 resistant strains and five susceptible strains of four major pest species (*D. saccharalis, H. armigera, H. zea*, and *O. nubilalis*) tested in five different laboratories support the dual model, but not the classical model. The 10 resistant strains are diverse in terms of how they were selected for resistance as well as their genetic basis and mechanism of resistance (Table S1). Both strains of *O. nubilalis* were selected with Cry1Ab protoxin in the laboratory^32^, the GA and GA-R strains were exposed to Bt crops in the field and selected with Cry1Ac protoxin in the laboratory^29^, the RR strain of *D. saccharalis* was selected with Bt corn leaf material in the laboratory, and the AR strain of *H. zea*^30^,^33^ as well as all four resistant strains of *H. armigera* tested here were selected with Cry1Ac activated toxin in the laboratory. In the four strains of *H. armigera*, resistance is caused by a recessive extracellular cadherin mutation in one strain (SCD-r1), a partially dominant intracellular cadherin mutation in another (SCD-r15), as well as partially dominant and dominant mutations affecting genes other than cadherin in the two remaining strains, respectively (SCD-423 and AY2)^34^,^35^,^36^,^37^. Reduced binding of Cry1Ac is associated with resistance in SCD-r1, but not in SCD-r15 where resistance entails interference with post-binding events^35^ (Table S1). Resistance in the Bt-RR strain of *D. saccharalis* is associated with reduced expression of three aminopeptidase N genes and cadherin, but not with mutations in the genes encoding these proteins^38^,^39^. In *O. nubilalis*, resistance is associated with reduced binding of toxin in the Europe-R strain, but not the RSTT-R strain^40^. In the AR and AR1 strains of *H. zea*, resistance is associated with increased alkaline phosphatase in the midgut lumen, but not with reduced binding of toxin^30^,^33^.

**Figure 1:**
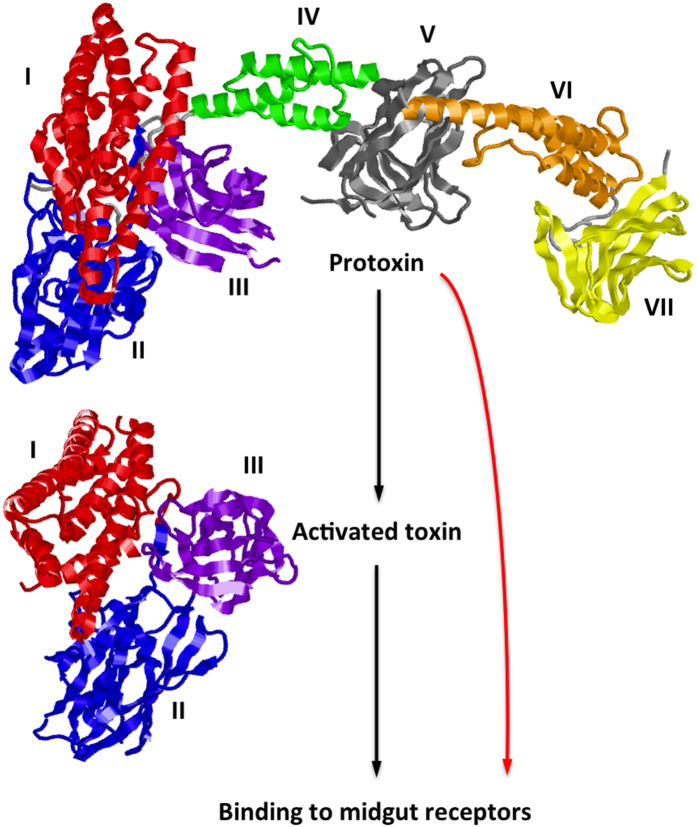
Bt protein mode of action. In the classical model (black arrows), inactive Cry1Ac protoxin (domains I-VII; PDB 4W8J) must be converted to activated toxin (domains I-III; PDB 4ARY) before binding to insect midgut receptors to exert toxicity. In the dual model, conversion of protoxin to activated toxin is the primary toxic pathway, but either intact protoxin or part of the protoxin other than the activated toxin also contribute to toxicity in a secondary toxic pathway (red arrow) that can be especially important in resistant insects with disruptions in the primary pathway, such as reduced binding of activated toxin to midgut receptors. In both models, binding to midgut receptors triggers post-binding events that eventually kill the insect.

The results summarized above imply that the classical model provides an adequate description for Bt toxin mode of action in susceptible insects, but apparently not in at least 10 resistant strains of insects. The evidence from both susceptible and resistant insects is consistent with the dual model as follows: In susceptible insects, conversion of protoxins to activated toxins is the primary toxic pathway, whereas intact protoxin or portions of protoxin other than the 65 kDa activated toxin cause mortality via a secondary pathway. However, in these 10 resistant strains, where the potency of activated toxins has been reduced by severe disruption of the primary toxic pathway, protoxins acting via a secondary pathway can be more potent than activated toxins (Fig. 1).

We considered an alternative hypothesis under which more of the data would fit the classical model: Reduced potency of activated toxin caused by removal of activated toxin from the toxic pathway (via degradation, sequestration, or excretion) is greater in resistant than susceptible insects when insects are fed activated toxin, but not when activated toxin is generated more gradually by conversion of protoxin within the insects. The second part of this hypothesis is essential to explain the observed patterns, because if enhanced removal also occurs for activated toxin generated by conversion of protoxin inside insects, this mechanism cannot explain why protoxins were more potent than activated toxins against 10 resistant strains. Although this alternative hypothesis is difficult to exclude in some cases, it does not explain the higher potency of protoxins than toxins in all of the 10 resistant strains described above. For example, in the resistant SCD-r1 and SCD-r15 strains of *H. armigera* tested in this study, the primary cause of resistance is mutant cadherin alleles *r1* and *r15*, respectively, which were introgressed into susceptible strain SCD^34^,^35^. Given that the major mechanism of resistance in these strains does not entail enhanced removal of activated toxin, the data do not support the alternative hypothesis for these strains. In addition, increased degradation of activated toxin was ruled out as a resistance mechanism in the Europe-R and RSTT-R strains of *O. nubilalis*^32^. In general, despite some evidence that increased degradation and sequestration can contribute to resistance^41^,^42^, these are not primary mechanisms of field-evolved resistance^27^,^28^. Moreover, we know of no data supporting the idea that any resistance mechanism increases removal of activated toxin when insects are fed activated toxin, but not when activated toxin is generated by conversion of protoxin within insects.

**Figure 2:**
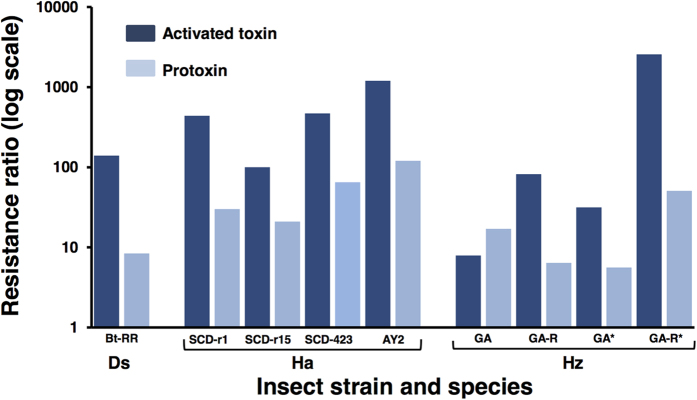
Resistance to the activated toxin (dark bars) and protoxin (light bars) of Bt proteins in seven strains of three species of insect pests. We tested Cry1Ab against *D. saccharalis* (Ds) strain Bt-RR and Cry1Ac against *H. armigera* (Ha) strains SCD-r1, SCD-r15, SCD-423, and AY2; and *H. zea* (Hz) strains GA and GA-R (Tables S1–S4). Resistance ratios are the concentration of activated toxin (or protoxin) killing 50% of larvae (LC_50_) for each resistant strain divided by the LC_50_ of activated toxin (or protoxin) for the conspecific susceptible strain. The asterisks after GA and GA-R (far right) indicate experiments where protoxin was activated with midgut juice from susceptible *H. zea* larvae; we used trypsin-activated protoxin in all other experiments (see Methods).

Despite the discrepancy between the classical model and the results from the 10 resistant strains of four species summarized above, both the dual and classical models correctly predict that reduced conversion of protoxin to activated toxin can yield higher resistance to protoxins than activated toxins, as seen in at least seven other resistant strains of five species: the KS-SC strain of *O. nubilalis*, the SERD5 strain of *Plutella xylostella*, the 198^r^ and Dpl^r^ strains of *Plodia interpunctella*, the Akola-R and LF5 strains of *H. armigera* and the MR strain of *Mythimna unipuncta*^23^,^24^,^25^,^26^,^27^,^28^,^43^,^44^. However, reduced conversion of protoxin to activated toxin is usually a relatively weak mechanism that is not common in field-selected strains, whereas reduced binding of toxin to midgut receptors confers high levels of resistance and is the most common field-selected mechanism of resistance to Bt toxins^27^,^28^. Aside from the 17 resistant strains discussed above, the responses to protoxins versus activated toxins remain to be evaluated in some other resistant strains that show reduced binding of toxin and a resistance ratio >500 for at least one Cry1A protoxin (e.g., the NO-QA strain of *P. xylostella*, the YHD2 strain of *Heliothis virescens*, the AZP-R strain of *P. gossypiella*, and the GLEN-Cry1Ac strain of *Trichoplusia ni*)^45^,^46^,^47^. The resistance to protoxin relative to activated toxin in these other strains may differ from the seven resistant strains studied here (Fig. 2, Tables S2–24) and the three strains of *O. nubilalis*^32^ and *H. zea*^30^ described above in which resistance ratios for Cry1A protoxins were at most 120.

**Figure 3:**
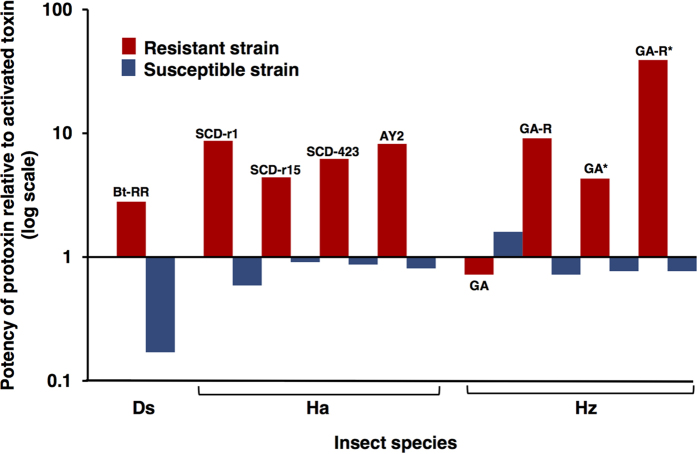
Potency of protoxin relative to activated toxin in seven resistant strains (red bars) and three susceptible strains (blue bars) of three species of insect pests. We tested Cry1Ab against *D. saccharalis* (Ds) and Cry1Ac against *H. armigera* (Ha) and *H. zea* (Hz) (Table S5). The potency of protoxin relative to activated toxin is the LC_50_ of an activated toxin divided by the LC_50_ of the corresponding protoxin. Values >1 indicate the protoxin was more potent than the activated toxin. Values <1 indicate the protoxin was less potent than the activated toxin. The asterisks after GA and GA-R (far right) indicate experiments where protoxin was activated with midgut juice from susceptible *H. zea* larvae; we used trypsin-activated protoxin in all other experiments (see Methods).

With a few notable exceptions^19^,^21^,^48^, previous work has largely ignored the potential binding to midgut receptors and contributions to toxicity of intact protoxins or portions of protoxins other than activated toxins. In addition, most studies of activated toxins have used mammalian trypsin or chymotrypsin to convert protoxin to activated toxin, which does not necessarily mimic activation by the mixture of proteases in larval midguts^17^,^21^. However, in one of the few previous direct comparisons for Cry1A proteins, mortality of susceptible *M. sexta* larvae was only slightly higher when Cry1Ab was activated with midgut juice from susceptible *M. sexta* larvae than with bovine trypsin^49^. In the comparisons here with the resistant GA and GA-R strains of *H. zea*, the potency of protoxin relative to activated toxin was lower for trypsin-activated Cry1Ac than Cry1Ac activated by midgut juice from susceptible *H. zea* larvae (Fig. 3 and Tables S4 and S5). These results with *H. zea* suggest that the data from resistant strains of other species tested only with trypsin-activated toxin may underestimate the potency of protoxins relative to activated toxins. Resistance to Cry1Ac protoxin of GA and GA-R relative to LAB-S was lower in this study (Fig. 2 and Table S4) than reported previously^29^. Factors that may have caused this pattern include differences between the two studies in the method of toxin preparation and the bioassay method (diet surface overlay here versus diet incorporation previously), and changes in the strains over time.

Although bioassay results indicate that in at least 10 resistant strains of four major lepidopteran pests, the intact protoxin or some part of the protoxin other than the activated toxin contributes to toxicity, much work remains to elucidate the details of the putative secondary toxic pathway. For both resistant strains of *O. nubilalis* mentioned above, *in vitro* experiments reveal that a substantial amount of protoxin remained intact after incubation with their larval midgut juice for 30 minutes at 30 °C^32^. After 60 minutes, the quantity of intact protoxin was markedly lower, but intermediate peptides larger than activated toxin and smaller than intact protoxin remained^32^. We hypothesize that the higher potency of protoxins relative to activated toxins against some resistant insects is caused by intact protoxin, peptides of intermediate size, or both. Although additional experiments are needed to distinguish between these possibilities, the available evidence from crystallography and bioassays, as well as *in vitro* assays of protein processing, binding, oligomerization and pore formation suggest that Bt protoxins contribute to insect mortality via a different toxic pathway than activated toxins.

Based on the prevailing perspective of the classical model of Bt mode of action, engineering plants to produce activated toxins rather than protoxins has been proposed as a way to defeat resistance mediated by reduced conversion of protoxins to activated toxins^24^,^26^. However, this approach eliminates any potential toxicity contributed by the putative secondary pathway, which might be particularly important against insects with reduced binding of activated toxins to midgut receptors that confers high levels of resistance to activated toxins. In addition, in a rare direct comparison based on a laboratory diet selection experiment, *H. zea* evolved resistance faster to activated toxin than protoxin^30^. Some cultivars of Bt crops produce activated toxins while others produce protoxins^3^,^50^. The findings reported here and some previous results^30^ suggest that Bt crops producing protoxins or both protoxins and activated toxins could be more effective and durable than those producing only activated toxins. The results also suggest that production of protoxins rather than activated toxins could be a natural bacterial strategy for delaying resistance in insects. For plants producing protoxins, the extent to which plant proteases convert protoxins to activated toxins may affect efficacy^51^. Better understanding of the higher potency of protoxins than activated toxins against some resistant insects may help to enhance the efficacy and durability of transgenic Bt crops.

## Methods

### Insect strains

We tested seven resistant strains and three susceptible strains of three lepidopteran species: *D. saccharalis, H. armigera*, and *H. zea* (Table S1). All susceptible strains were reared in the lab without exposure to Bt toxins or other insecticides.

#### Diatraea saccharalis

The susceptible strain (Bt-SS) was established using larvae collected from corn fields near Winnsboro in northeastern Louisiana during 2004. A Bt-resistant strain (Bt-RR) was developed from a single isoline family using an F_2_ screen^52^. Bt-RR larvae completed development on commercial Cry1Ab corn hybrids^52^. Before the current study, the Bt-RR strain was backcrossed three times with the Bt-SS strain and reselected for resistance with Cry1Ab corn leaf tissue in the F_2_ generation after each backcross.

#### Helicoverpa armigera

The susceptible strain of *H. armigera* (SCD) originated from the Cote D’Ivoire in the 1970s and was obtained from Bayer CropScience in 2001. We tested four previously described resistant strains of *H. armigera* (SCD-r1, SCD-r15, SCD-423 and AY2) that were each derived from northern China and selected by exposing larvae in the laboratory to diet overlaid with Cry1Ac activated toxin^34^,^35^,^36^,^37^,^53^.

SCD-r1 and SCD-r15 were created by introgressing resistant cadherin alleles *r1* and *r15*, respectively, into the susceptible SCD strain via repeated backcrossing and selection, as described by Yang *et al.*^34^ for SCD-r1. The recessive *r1* allele has a premature stop codon that disrupts the extracellular region of cadherin and interferes with binding of Cry1Ac^34^. The non-recessive *r15* allele has a deletion of 55 amino acids disrupting the intracellular region of cadherin, which is not directly involved with binding, but disrupts post-binding events^35^. The *r1* allele was derived from the GYBT strain, which was obtained from the GY strain by 28 generations of selection with Cry1Ac^53^. The GY strain was started in August 2001 with 300 larvae collected from late season Bt cotton in Gaoyang County, Hebei Province, China^53^. The *r15* allele was derived from the AY-r15 strain, which was started from a single male moth that was collected from a light trap in June 2009 from Anyang County, Henan Province and mated with a virgin female from SCD-r1^35^. Homozygosity for *r15* in AY-r15 was achieved with a series of crosses and screening in which single-pair families were selected for the presence of the *r1*5 allele with PCR and for resistance to Cry1Ac^35^. The resistant SCD-423 strain was derived from the AY423 strain. AY423 had non-recessive resistance that was not conferred by a cadherin mutation^36^. AY423 was started from a single male moth collected from a light trap in June 2009 from Anyang County and mated with a virgin female from SCD^36^. The resistance in AY423 was introgressed into SCD with repeated crosses to SCD followed by selection with Cry1Ac^36^. The resistant AY2 strain had dominant resistance to Cry1Ac^37^. Male and female moths (n = 222) were collected from light traps at Anyang in June 2011 and allowed to mate among themselves. The resulting F_1_ progeny were screened for resistance at a diagnostic concentration of Cry1Ac^37^. We started resistant strain AY2 from a single-pair family generated by crossing one of the resistant F_1_ males from Anyang with a virgin female from the susceptible SCD strain. For the next 10 generations, larvae from AY2 were selected with increasing concentrations of Cry1Ac^37^.

#### Helicoverpa zea

We used three previously described strains of *H. zea*: a susceptible laboratory strain (LAB-S) obtained from Benzon Research Inc. (Carlisle, PA) and two strains from Georgia (GA and GA-R) with significant resistance to Cry1Ac relative to LAB-S^29^. GA was started with 180 larvae collected on Cry1Ab corn from Tifton, Georgia in 2008 and was reared in the laboratory without exposure to Bt toxins for >40 generations before this study was conducted. GA-R was derived from GA and selected in the laboratory with Cry1Ac protoxin in diet^29^. In two experiments conducted before the current study, survival on Bt cotton producing Cry1Ac was 0 and 3.3% for GA, compared with 11.7 and 27% for GA-R, indicating that GA had little or no resistance to Bt cotton producing Cry1Ac^29^,^54^.

### Bioassays

All bioassays were done in the laboratory using established techniques with larvae tested individually on diet with a series of 5 to 8 concentrations of protoxin and activated toxin, including controls with no protoxin or activated toxin. The mean number of larvae tested in 34 bioassays for each combination of insect strain and protoxin or activated toxin was 339 (range = 189 to 744, total = 11,520, Tables S2–S4). Details for sources of protoxins, activated toxins, and bioassay methods for each species are provided below.

#### D. saccharalis

We used diet incorporation bioassays to test Cry1Ab protoxin and activated toxin against neonates (<24 h old)^55^. Mario Soberón and Alejandra Bravo provided Cry1Ab protoxin that was produced by Bt cells and purified as described previously^56^. Trypsin-activated Cry1Ab was purchased from Marianne Pusztai-Carey at Case Western Reserve University, who obtained Cry1Ab protoxin from *E. coli* cells transformed with the *cry1Ab* gene from the HD1 strain of Bt subsp. *kurstaki*^57^. The inclusion bodies were solubilized at pH 10.5 in the presence of a reducing agent. The Cry1Ab protoxin was digested by commercial bovine trypsin and purified by anion exchange HPLC. The fractions containing toxin were analyzed by gel filtration HPLC and SDS-PAGE, desalted and lyophilized^57^. Cry1Ab toxin and activated toxin were diluted with distilled water and mixed with diet. We added ca. 0.7 ml of diet into each well of a 32-well plate and put one neonate in each well. After 7 days at 28 °C, 16L:8D, and 50% RH, larvae were scored as dead if they died or if they weighed ≤0.1 mg based on visual estimation.

#### H. armigera

We used diet surface overlay bioassays to test Cry1Ac protoxin and activated toxin against second instars that had been starved for 4 hours^34^. Cry1Ac protoxin and trypsin-activated toxin were purchased from Marianne Pusztai-Carey, who obtained Cry1Ac protoxin from *E. coli* cells transformed with the *cry1Ac* gene from the HD1 strain of Bt subsp. *kurstaki*. She produced trypsin-activated Cry1Ac using the same method as described above for Cry1Ab^57^. Cry1Ac protoxin and activated toxin were diluted with a phosphate buffer solution (PBS; 0.01 M, pH 7.4). We put 900 μl of liquid artificial diet in each well of a 24-well plate. After the diet cooled and solidified, 100 μl of PBS with an appropriate concentration of protoxin or activated toxin was added to each well and allowed to air dry. We put one second instar in each well. After 5 days at 26 ± 1 °C, 16L:8D, and 60% RH, larvae were scored as dead if they died or if they weighed <5 mg.

#### H. zea

We used diet surface overlays^58^ to test neonates (<18 h old) against Cry1Ac protoxin and toxin activated with either trypsin or midgut extract from susceptible *H. zea* larvae. We dispensed 750 μl of a bean-based diet into each well of a 128-well bioassay tray^29^. After the diet cooled and solidified, we added to each well 50 μl of a dilution containing 0.1% Triton X-100 and the appropriate concentration of protoxin or activated toxin. After the bioassay tray was rotated for 1 h to allow the dilution to spread evenly and dry, we put one neonate in each well and covered each tray with a ventilated plastic cover. We recorded mortality after 7 days at 27 ± 1 °C, 14L:10D, and 60 ± 10% RH.

We conducted three sets of bioassays with *H. zea.* In all three sets, we used Cry1Ac protoxin provided by Jie Zhang. He obtained Cry1Ac protoxin by growing the HD73 strain of Bt subsp. *kurstaki* in 1/2 Luria-Bertani medium using the repeated crystal solubilization method^59^. In the first and second sets of bioassays, we compared Cry1Ac protoxin with trypsin-activated Cry1Ac purchased from Marianne Pusztai-Carey that she produced as described above. In the third set, we compared Cry1Ac protoxin with Cry1Ac activated by midgut extract as follows: We chilled five fourth instars of the susceptible LAB-S strain of *H. zea* on ice, removed their midguts, and put the midguts in ice cold NaCl (0.15 M) solution. Each midgut was ground by hand with a glass homogenizer and the soluble extract was separated as the supernatant after centrifuging at 100,000 g for 10 min at 4 °C. with BSA as the standard to measure the concentration of protein in the midgut extract and Cry1Ac protoxin. Cry1Ac protoxin was incubated with midgut extract (25 protoxin:1 extract by weight) at 37 °C for 6 h. Cry1Ac activated toxin was precipitated by adding acetic acid to adjust pH to 4.5, followed by centrifugation at 10,000 g for 10 min. The pellet was washed three times using ice cold double distilled water, dissolved in 50 mM pH 10.0 Na_2_CO_3_ with 0.01 mM dithiothreitol and stored at −20 °C. We used the Bradford method^60^ with BSA as the standard to measure the concentration of protein in the midgut extract, and the concentrations of Cry1Ac protoxin and activated toxin.

### Analysis of data and crystal structures

Supplementary Methods provides details of the analysis of the data and the crystal structures of Cry1Ac protoxin and activated toxin.

## Additional Information

**How to cite this article**: Tabashnik, B. E. *et al.* Dual mode of action of Bt proteins: protoxin efficacy against resistant insects. *Sci. Rep.* **5**, 15107; doi: 10.1038/srep15107 (2015).

## Supplementary Material

Supplementary Information
