# Supplementary material for: Dual mode of action of Bt proteins: protoxin efficacy against resistant insects
Source: Sci Rep. 2015 Oct 12;5:15107. doi: 10.1038/srep15107 (PMC4601037; doi:10.1038/srep15107)
Supplement: Supplementary Information [file srep15107-s1.pdf]

Supplementary Information

Dual Mode of Action of Bt Proteins: Protoxin Efficacy Against Resistant Insects

Bruce E. Tabashnik[1], Min Zhang[1], Jeffrey A. Fabrick[2], Yidong Wu[3], Meijing Gao[3], Fangneng Huang[4], Jizhen Wei[1,5], Jie Zhang[5], Alexander Yelich[1], Gopalan C. Unnithan[1], Alejandra Bravo[6], Mario Soberón[6], Yves Carrière[1] & Xianchun Li[1]

[1]Department of Entomology, University of Arizona, Tucson, Arizona, USA.
[2]U.S. Department of Agriculture, Agricultural Research Service, U.S. Arid Land Agricultural Research Center, Maricopa, Arizona, USA.
[3]Department of Entomology, College of Plant Protection, Nanjing Agricultural University, Nanjing 210095, China.
[4]Department of Entomology, Louisiana State University Agricultural Center, Baton Rouge, Louisiana, USA.
[5] State Key Laboratory for Biology of Plant Diseases and Insect Pests, Institute of Plant Protection, Chinese Academy of Agricultural Sciences, Beijing, China.
[6]Instituto de Biotecnología, Universidad Nacional Autónoma de México, Cuernavaca, Morelos, Mexico.

This Supplementary Information contains:
Supplementary Methods
Supplementary Tables S1-S5
Supplementary Figure S1

Supplementary Methods

Statistical analysis. We used probit analysis of mortality data to estimate the concentration of toxin killing 50% of larvae (LC_{50}) and its 95% fiducial limits (FL), as well as the slope of the concentration-mortality line and its standard error (SE). Probit analysis was done with SAS [61] for *D. saccharalis*, PoloPlus [62] for *H. armigera*, and POLO PC [62] for *H. zea.*

We calculated the resistance ratio as the LC_{50} for a resistant strain divided by the corresponding LC_{50} for the conspecific susceptible strain. We considered values of LC_{50} significantly different if their 95% FL did not overlap, which is a conservative criterion [64]. We calculated the reduction in resistance ratio for protoxins relative to activated toxins as the resistance ratio for the protoxin divided by the resistance ratio for the corresponding activated toxin. Potency is inversely related to LC_{50} [31]. We calculated the potency of protoxin relative to activated toxin as the LC_{50} of the activated toxin divided by the LC_{50} of the corresponding protoxin [31].

We used paired t-tests [65] of log-transformed data to determine if the resistance ratio differed between protoxins and their corresponding activated toxins, and if potency of protoxins relative to activated toxins differed between resistant and susceptible strains. We used one-sample t-tests [65] of log-transformed data to determine if the potency of protoxins relative to activated toxins against susceptible strains and resistant strains differed from the predicted value of 0.50. We used linear regression [65] of log-transformed data to determine if the reduction in the resistance ratio for protoxin relative to activated toxin was associated with the resistance ratio for activated toxin. We used t-tests [65] of log-transformed data to determine if the source of protoxin relative to activated toxin affected the reduction in resistance ratio for protoxin relative to activated toxin and the potency of protoxin relative to activated toxin (see below).

In six of the nine sets of bioassay data used to compare responses to protoxins versus activated toxins (Methods), we obtained the activated toxin by activating the protoxin used in the bioassays: all bioassays with the four resistant strains of *H. armigera* and one set of bioassays with each of the two resistant strains of *H. zea.* In the other three sets of bioassays, the activated toxin and protoxin were from different sources: one set of bioassays with *D. saccharalis* and with each of the two resistant strains of *H. zea.* We found no significant difference in the reduction in resistance ratio for protoxin relative to activated toxin (Tables S2-S4) between the six data sets with the same source of protoxin and activated toxin (back-transformed mean =11) and the three data sets with different sources for protoxin and activated toxin (back-transformed mean = 4.7) (t-test of log-transformed data, df = 7, t = 0.89, P = 0.40). Likewise, we found no significant difference in the potency of protoxin relative to activated toxin (Table S5) between the six data sets with the same source of protoxin and activated toxin (back-transformed mean =8.3) and the three data sets with different sources for protoxin and activated toxin (back-transformed mean = 2.6) (t-test of log-transformed data, df = 7, t =1.7, P = 0.14). Because the source of the protoxin relative to activated toxin had no significant effect on either reduction in resistance ratio or potency of protoxin relative to activated toxin, the results reported are based on all nine sets of data. This approach is conservative because support for the dual model is numerically stronger if the three data sets with different sources of protoxin and activated toxin are excluded.

Crystal structures: Cry1Ac protoxin and activated toxin. We downloaded structures for Cry1Ac protoxin (4W8J) and activated toxin (4ARY) from the Protein Data Bank (PDB) and visualized them using RasMol 2.7.5.2 (RasWin Molecular Graphics) [66]. Structure 4W8J is for Cry1Ac-Δ14C, which corresponds to amino acids Thr34-Glu1178 of full-length Cry1Ac protoxin, but with the final 14 cysteine residues converted to serine (Cys to Ser at amino acids 661, 730, 796, 802, 814, 816, 822, 837, 990, 1025, 1045, 1063, 1076 and 1125). Structure 4ARY for Cry1Ac activated toxin corresponds to Thr31-Thr611 of 4W8J, except for Phe148Leu and Phe462Val substitutions. For activated toxin, Fig. 1 shows domain I as amino acids 31-258, domain II as 259-463, and domain III as 464-611. For protoxin, Fig. 1 shows each domain with its amino acids as:
I: 35-247, II: 258-458, III: 470-608, IV: 610-682, V: 701-872, VI: 875-976, and VII: 991-1176.

References

61. SAS Institute. SAS/STAT 9.3 User's Guide, SAS Institute, Cary, NC, USA (2010).
62. LeOra Software. PoloPlus: A User's Guide to Probit and Logit Analysis, Berkeley, CA, USA (2003).
63. LeOra Software. POLO-PC: A User's Guide to Probit and Logit Analysis, Berkeley, CA, USA (1987).
64. Tabashnik, B.E., Cushing, N.L. & Johnson, M.W. Diamondback moth (Lepidoptera: Plutellidae) resistance to insecticides in Hawaii: Intra-island variation and cross-resistance. *J. Econ. Entomol.* **80**, 1091-1099 (1987).
65. VassarStats: Website for Statistical Computation. http://faculty.vassar.edu/lowry/VassarStats.html.
66. Sayle, R. & Milner-White, E.J. RasMol: biomolecular graphics for all. *Trends Biochem. Sci.* **20**, 374-376 (1995).

Supplementary Table S1. Seven resistant strains and three susceptible strains of three species of major lepidopteran pests tested in this study with protoxins and activated toxins.

Insect species	Strain name	Strain type[a]	Mechanism/genetic basis of resistance	Reference(s)
D. saccharalis	Bt-RR	R	non-recessive, reduced expression of cadherin and three amino-peptidases N	37, 38, 52, 55, 67
	Bt-SS	S		
H. armigera	SCD-r1	R	reduced binding, recessive mutation affecting extracellular domain of cadherin	33, 53
	SCD-r15	R	disruption of post-binding events, non-recessive mutation affecting intracellular domain of cadherin	34
	SCD-423	R	non-recessive, not cadherin mutation	35
	AY2	R	dominant, not cadherin mutation	36
	SCD	S		
H. zea	GA-R	R	non-recessive, mechanism not known	29
	GA	R	non-recessive, mechanism not known	29
	LAB-S	S		

[a]R: resistant, S: susceptible

Reference

67. Ghimire, M.N., Huang, F., Leonard, R., Head, G.P. & Yang, Y. Susceptibility of Cry1Ab-susceptible and -resistant sugarcane borer to transgenic corn plants containing single or pyramided *Bacillus thuringiensis* genes. *Crop Prot.* **30**, 74-81 (2011).

Supplementary Table S2. Reduction in resistance ratio for Cry1Ab protoxin relative to Cry1Ab activated toxin against *Diatraea saccharalis* based on responses of a resistant strain (Bt-RR) relative to a susceptible strain (Bt-SS).

Strain[a]	Toxin form	n	Slope (SE)	LC_{50} (95% FL)[b]	Resistance ratio (RR)[c]	Reduction in RR[d]
Bt-RR	Activated	504	1.3 (0.1)	18.2 (14 – 25)	140	
Bt-SS	Activated	744	1.0 (0.1)	0.129 (0.08 – 0.20)		
Bt-RR	Protoxin	482	2.2 (0.2)	6.47 (5.4 – 7.7)	8.4	17
Bt-SS	Protoxin	711	1.2 (0.09)	0.774 (0.58 – 1.0)		

[a] See **Supplementary Table S1** for strain details.

[b] Concentration causing death or no significant weight gain in 50% of larvae ("practical mortality") and its 95% fiducial limits in μg Cry1Ab per g diet

[c] LC_{50} of resistant strain Bt-RR divided by LC_{50} of susceptible strain Bt-SS

[d] Resistance ratio of activated toxin divided by resistance ratio of protoxin

Supplementary Table S3. Reduction in resistance ratio for Cry1Ac protoxin relative to Cry1Ac activated toxin against *Helicoverpa armigera* based on responses of four resistant strains (SCD-r1, SCD-r15, SCD-423, AY2) relative to a susceptible strain (SCD).

Strain [a]	Toxin form	n	Slope (SE)	LC_{50} (95% FL) [b]	Resistance ratio (RR) [c]	Reduction in RR [d]
SCD-r1	Activated	240	1.4 (0.2)	9.70 (7.0 – 13)	440	
SCD	Activated	480	1.4 (0.2)	0.022 (0.013 – 0.033)		
SCD-r1	Protoxin	231	1.9 (0.3)	1.12 (0.72 – 1.5)	30	15
SCD	Protoxin	189	2.4 (0.3)	0.037 (0.030 – 0.045)		
SCD-r15	Activated	288	1.4 (0.2)	3.20 (2.0 – 6.4)	100	
SCD	Activated	288	2.0 (0.2)	0.032 (0.025 – 0.041)		
SCD-r15	Protoxin	240	2.2 (0.3)	0.73 (0.47 – 1.8)	21	4.8
SCD	Protoxin	240	2.2 (0.3)	0.035 (0.028 – 0.043)		
SCD-423	Activated	288	1.4 (0.2)	12.2 (8.7 – 17)	470	
SCD	Activated	336	1.6 (0.2)	0.026 (0.020 – 0.034)		
SCD-423	Protoxin	288	1.4 (0.2)	1.96 (0.63 – 3.4)	65	7.2
SCD	Protoxin	240	2.3 (0.3)	0.030 (0.023 – 0.037)		
AY2	Activated	336	1.4 (0.3)	51.0 (35 – 91)	1200	
SCD	Activated	336	2.0 (0.2)	0.042 (0.035 – 0.051)		
AY2	Protoxin	336	1.4 (0.2)	6.24 (4.8 – 8.8)	120	10
SCD	Protoxin	336	1.7 (0.2)	0.052 (0.041 – 0.066)		

[a] See **Supplementary Table S1** for strain details.

[b] Concentration killing 50% of larvae and its 95% fiducial limits in μg Cry1Ac per cm^2 diet

[c] LC_{50} of resistant strain divided by LC_{50} of susceptible strain

[d] Resistance ratio of activated toxin divided by resistance ratio of protoxin

Supplementary Table S4. Reduction in resistance ratio for Cry1Ac protoxin relative to Cry1Ac activated toxin against *Helicoverpa zea* based on responses of two resistant strains (GA and GA-R) relative to a susceptible strain (LAB-S).

Strain [a]	Toxin form	n	Slope (SE)	LC_{50} (95% FL) [b]	Resistance ratio (RR) [c]	Reduction in RR [d]
GA-R	Activated	237	0.88 (0.2)	5320 (1900 – 48,000)	82	
LAB-S	Activated	333	2.1 (0.3)	64.8 (48 – 82)		
GA-R	Protoxin	271	0.63 (0.1)	582 (9.0 – 2200)	6.4	13
LAB-S	Protoxin	335	1.3 (0.2)	90.6 (65 – 130)		
GA	Activated	384	1.9 (0.4)	707 (510 – 1100)	7.9	
LAB-S	Activated	279	1.8 (0.3)	89.8 (60 – 210)		
GA	Protoxin	381	1.4 (0.3)	987 (640 – 2100)	17	0.46
LAB-S	Protoxin	282	1.6 (0.3)	57.8 (33 – 290)		
GA-R	Activated [e]	329	0.63 (0.2)	264,000 (60,000– 210,000)	2600	
GA	Activated [e]	324	1.4 (0.2)	3230 (2100 – 6500)	31	
LAB-S	Activated [e]	283	1.4 (1)	103 (73 – 150)		
GA-R	Protoxin	329	0.89 (0.1)	6780 (2300– 56,000)	51	51
GA	Protoxin	333	1.2 (0.1)	756 (530 – 1100)	5.6	5.5
LAB-S	Protoxin	287	1.2 (0.2)	134 (90 – 230)		

[a] See **Supplementary Table S1** for strain details.

[b] Concentration killing 50% of larvae and its 95% fiducial limits in µg Cry1Ac per cm^2 diet

[c] LC_{50} of resistant strain divided by LC_{50} of susceptible strain

[d] Resistance ratio of activated toxin divided by resistance ratio of protoxin

[e] Activated with midgut juice from LAB-S larvae, all others activated with trypsin (see Methods for details)

Supplementary Table S5. Potency ratio of Bt protoxins relative to activated toxins for resistant and susceptible strains of three major crop pests, calculated as the LC_{50} of an activated toxin divided by the LC_{50} of the corresponding protoxin (see **Supplementary Tables S2-S4**). Values >1 indicate the protoxin was more potent than the activated toxin, which occurred in all cases for resistant strains except for trypsin-activated Cry1Ac against the GA strain of *H. zea.* Values <1 indicate the activated toxin was more potent than protoxin, which occurred in all cases for susceptible strains, except in one of two tests of the susceptible LAB-S strain of *H. zea* against trypsin-activated Cry1Ac.

Species [a]	Resistant strain name	Potency ratio (protoxin relative to activated toxin)	
		Resistant strain	Susceptible strain
D. saccharalis	Bt-RR	2.8	0.17
H. armigera	SCD-r1	8.7	0.59
	SCD-r15	4.4	0.91
	SCD-423	6.2	0.87
	AY2	8.2	0.81
H. zea	GA	0.72	1.6
	GA-R	9.1	0.72
	GA[b]	4.3	0.77
	GA-R[b]	39	0.77
Mean[c]		5.7	0.70[d]

[a] Cry1Ab was tested against *D. saccharalis*, Cry1Ac against *H. armigera* and *H. zea*

[b] Activated with midgut juice from susceptible LAB-S larvae, all others activated with trypsin (see Online Methods for details)

[c] Back-transformed mean of the log-transformed data

[d] The mean for susceptible strains is based on one pair of bioassays for the Bt-SS strain of *D. saccharalis*, four pairs of bioassays for the SCD strain of *H. armigera*, and three pairs of bioassays for the LAB-S strain of *H. zea* (**Supplementary Tables S2-S4**, respectively).

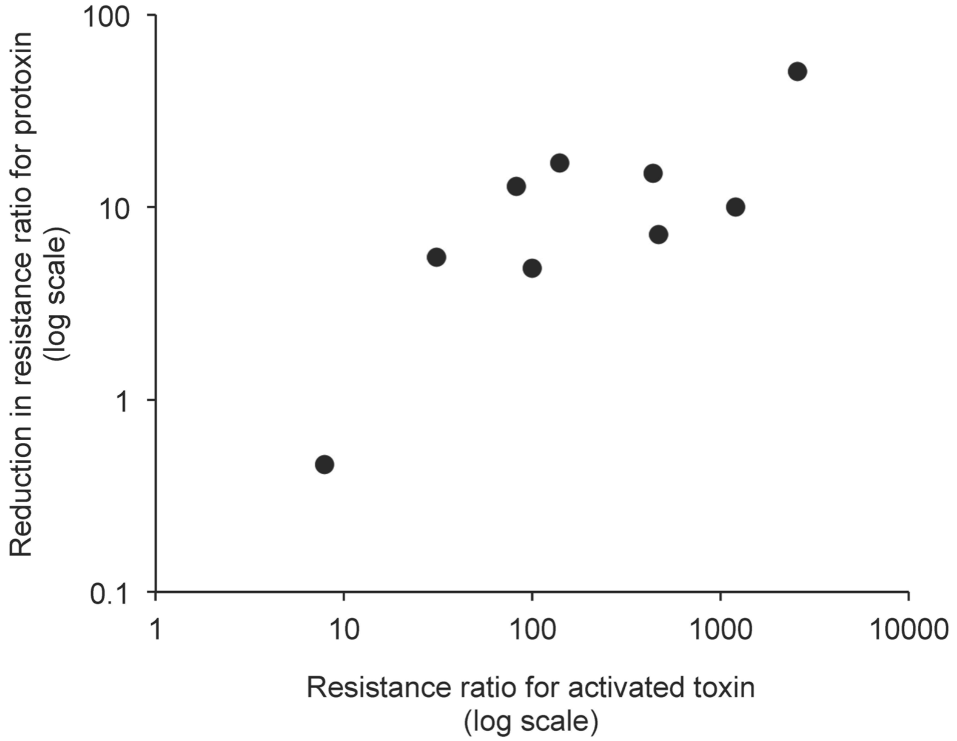

Supplementary Figure S1. Association between reduction in resistance ratio for protoxin relative to activated toxin and resistance ratio for activated toxin. We calculated the resistance ratio as the concentration of activated toxin (or protoxin) killing 50% of larvae (LC_{50}) for a resistant strain divided by the LC_{50} of activated toxin (or protoxin) for a conspecific susceptible strain (**Supplementary Tables S2-S4**). We measured the reduction in resistance ratio for the protoxin relative to its activated toxin counterpart as the resistance ratio for the activated toxin divided by the resistance ratio for the corresponding protoxin. The reduction in resistance for protoxin relative to activated toxin was significantly associated with the resistance ratio for activated toxin (regression of log-transformed data, $r^2 = 0.71$, df = 7, P = 0.004).
